# Changes in Secondary Healthcare Use Over Retirement Transition: Examining Social Differences With Swedish Register Data

**DOI:** 10.3389/fsoc.2022.737595

**Published:** 2022-03-28

**Authors:** Martin Wetzel, Stefanie König, Susanne Kelfve

**Affiliations:** ^1^Institute of Sociology and Social Psychology, University of Cologne, Cologne, Germany; ^2^Department of Psychology and Centre for Ageing and Health - AgeCap, University of Gothenburg, Gothenburg, Sweden; ^3^Division Ageing and Social Change, Department of Culture and Society, Linköping University, Linköping, Sweden; ^4^Aging Research Center, Karolinska Institutet & Stockholm University, Stockholm, Sweden

**Keywords:** retirement, secondary healthcare, socio-economic status, gender, Sweden, register data

## Abstract

**Background:**

Despite its relevance for healthcare expenditures and public health, few studies have examined how secondary healthcare use changes during the retirement transition. We therefore use Swedish register data to examine whether retirement is associated with intensified secondary healthcare use overall and for specific subgroups based on gender and education.

**Methods:**

The sample was all individuals registered in Sweden who retired from paid work in 2010. We used Generalised Estimating Equations models to analyse changes in two indicators of secondary healthcare use, namely specialist visits and hospitalisation, from 3 years prior to 5 years after retirement.

**Results:**

Retirement is not associated with changes in specialist visits or hospitalisation *per se*. Three years before retirement, women were more likely to visit a specialist but less likely to be hospitalised than men; these gender differences disappeared approximately 1 year before retirement. Women with high education were more likely to visit a specialist than women with low education across the entire retirement transition, particularly post-retirement. Significant differences with regard to specialist visits between male educational groups only emerged 12 months after retirement. There were no educational differences with regard to hospitalisation.

**Conclusions:**

We conclude that secondary healthcare use in Sweden does not generally change with retirement. However, over the course of retirement gender differences in secondary healthcare use tend to decrease and within-gender educational differences tend to increase. We interpret the results as reflecting the role of labour market institutions in contributing to gender differences but repressing educational differences in secondary healthcare use.

## Introduction

Research on how healthcare use changes during retirement—overall and for specific subgroups—has the potential to inform healthcare budgets, identify social inequalities and vulnerable groups, and contribute to a better understanding of how retirement affects individuals' wellbeing in later life. Little is, however, currently known about how secondary healthcare use changes during the retirement transition. In the current study we therefore use Swedish register data to examine how two indicators of secondary healthcare use, namely specialist visits and hospitalisation, change during retirement and how retirement is interrelated with differences between men and women with different educational backgrounds. We focus specifically on secondary healthcare use because it is associated with more serious health problems and is also often more expensive than primary healthcare (e.g., general practitioner visits).

There are several reasons why secondary healthcare use might change with retirement. For one, by freeing up time, retirement changes people's opportunity structures for health behaviour (Olds et al., 2018). For instance, with retirement, people seem to engage more often in physical activities (Stenholm et al., 2016). However, the effect is unequally distributed with people of higher socio-economic status (SES) engaging stronger in activities while those with lower SES tend to reduce their activities (Barnett et al., 2012). At the same time, retirement assumingly reduces work- and transport-related physical activity (Xue et al., 2020). For other health behaviours, as smoking, drinking and dietary behaviour, the findings are inconclusive (Helldán et al., 2012; Si Hassen et al., 2017; Xue et al., 2020). Another line of arguments why retirement might affect secondary healthcare use, is that retirement directly affect health (and thus on healthcare use). This has been long-time for debate with equally inconclusive results (for overviews, Gallo, 2013; van der Heide et al., 2013; Xue et al., 2020). Most recent studies suggest that health develops non-linearly during retirement with differences in the anticipatory, short- and long-term effects of retirement (Westerlund et al., 2009; Wetzel et al., 2016; Schmälzle et al., 2019) and that health develops differences over SES (Westerlund et al., 2009). In sum, there are reasons why retirement might increase secondary healthcare use (due to e.g., more time for doctoral visits), but also decrease (due to e.g., more time for healthy lifestyle), and also reason to believe that the pattern of change might depend on the SES and time frame.

So far, several studies have empirically examined how secondary healthcare use changes during retirement with inconclusive results. A Swedish study based on full population data found no evidence that hospitalisation changed with retirement (Hagen, 2018) and another Swedish study shows that over the course of retirement (age 62–70) secondary healthcare increases however depending on the pathway (e.g., early vs. late retirement, bridge employment) individuals follow (König et al., 2021). A Danish register-based study found that hospitalisation due to mental disorder increased before retirement but then decreased after retirement (Olesen et al., 2015) while another Danish study showed that statutory retirement had no effect on hospitalisation (Nielsen, 2019). Other studies from central Europe and the United States found that neither specialist visits (Bíró, 2016; Lucifora and Vigani, 2018) nor nights spent in hospital (Coe and Zamarro, 2015; Eibich, 2015; Grøtting and Lillebø, 2020) changed with retirement, and that doctor visits (all types) either decreased (Coe and Zamarro, 2015; Eibich, 2015; Frimmel and Pruckner, 2020) or increased (Bíró, 2016; Lucifora and Vigani, 2018). In China, doctor visits (all types) and hospitalisation both appear to increase with retirement (Zhang et al., 2018).

Summing up, the existing literature on secondary healthcare use during retirement is inconclusive. An important caveat of previous studies is, however, that most studies have assessed only the population average, while just a few have examined potential differences between social groups (e.g., Eibich, 2015; Olesen et al., 2015; Bíró, 2016; Bíró and Elek, 2018; Zhang et al., 2018; Grøtting and Lillebø, 2020; König et al., 2021). How secondary healthcare use changes during retirement might depend, however, on gender and/or SES. Many studies indicate that women use more healthcare than men, which seems to be at least partly explained by gender differences in health (Green and Pope, 1999). Even though women tend to live longer and have fewer life-threatening conditions (e.g., heart attacks), they also tend to have more non-fatal chronic conditions and mobility limitations than men which may increase their secondary healthcare use (Read and Gorman, 2010). Women also experience greater physiological and hormonal changes over the life course (e.g., menopause) than men. Whether the magnitude of gender differences in secondary healthcare use changes with retirement has not yet been answered. It is, however, widely acknowledged that men and women's experiences in the labour market differ dramatically, which seems likely to affect their experience of retirement in ways that are relevant for their secondary healthcare use (Moen, 1996). For instance, women tend to occupy fewer positions of power, receive less income, and are more likely to work part-time, and hence have less access to some health-related resources, more chronic stressors, and also more time to access healthcare relative to men (Read and Gorman, 2010). Retirement may at least partially level out any gender differences in secondary healthcare use stemming from gendered experiences in the labour market.

For both men and women, trajectories of secondary healthcare use during the retirement transition may also differ across SES groups. Lower SES groups typically have worse health than higher SES groups across the entire life course (Mirowsky and Ross, 2017), and there is some evidence that health disparities across SES groups increase after retirement (Schaap et al., 2018). The observed increase in health disparities suggests that SES-related differences in secondary healthcare use may likewise increase during the retirement transition. In studies that examined how retirement affected secondary healthcare use in different SES groups, one study found no difference between income groups in Sweden (Hagen, 2018), the other found that hospitalisation increased more for people with less education in China (Zhang et al., 2018) and the third found a decrease in hospital days only for male blue collar workers but not for white collar workers (Frimmel and Pruckner, 2020). Similarly, a forth study finds a decrease in the likelihood for hospitalisation only for low SES groups (Grøtting and Lillebø, 2020).

In the current study we assess changes in specialist visits and hospitalisation across the retirement transition overall and separately for men and women with different educational backgrounds. Since selectivity is a problem in longitudinal studies on health (Lynch, 2003), the current study uses Swedish register data for an entire birth cohort and hence is based on a full, unselected data. Healthcare in Sweden is publicly funded and provided to all citizens for free (children) or at a regulated low cost (adults). Patients can access secondary healthcare through referral from a primary care provider, or they can contact specialists directly. To examine changes potentially preceding retirement as well as short- and long-term health trajectories during the retirement transition, we examine secondary healthcare use over the course of 3 years prior through 5 years after retirement. We focus on old-age retirees coming from work only. Although this is not the most often applied retirement pathway in Sweden representing only about 24% of all recent retirement transitions (König et al., 2021), at this pathway experienced changes in daily time structures and resources should be most pronounced (see Schmälzle et al., 2019 for a similar argument).

To sum up, our study contributes to previous research in five ways. First, our article focuses on different subgroups of individuals, rather than looking at a mean effect. Second, our study examines also long- and short-term changes across retirement, which is a benefit in contrast to rather direct effects measured by other approaches such as instrumental variable approaches or a regression discontinuity design. Looking at healthcare use on a monthly basis allows us to differentiate between changes that precede retirement and changes that follow the retirement transitions. Although this approach is descriptive in nature, insights about the succession of changes can be derived. Third, we apply a rather strict definition of retirement which provides a clearer picture compared to previous studies where phased retirement and stepwise reduction of work is mixed with transitions from work to full retirement. Fourth, we use two measures of healthcare use which allows us to detect potential differences between healthcare seeking behaviour and healthcare needs. Last, we use register data which has a clear advantage compared to survey data which may be limited by sample selection.

## Methods

### Data Sources

Data on retirement, gender and education were from the Longitudinal Integration Database for Health Insurance and Labour Market Studies (LISA) database. LISA is a Swedish register including information on income from different sources and socio-demographic factors, collected and administered by Statistics Sweden. To identify the month of retirement, we used information from the “Activity Register” for 2010, a register also administered by Statistics Sweden which includes monthly information on employment status.

Data on specialist visits and hospitalisation were from the National Patient Register. Both registers are administrated by the Swedish National Board of Health and Welfare. The hospitalisation registry includes more than 99% of all inpatient hospital discharges (Ludvigsson et al., 2011).

### Study Population and Sample

Our study population was all individuals registered in Sweden and born between 1943 and 1949 who were gainfully working in 2009 and retired in 2010 (the last year in our data that allows for 5 years follow-up)^1^. In 2009, a total of 963,030 individuals were aged between 60 and 66 years (inclusive) and thus eligible for pension payments in 2010. Of those, 843,212 were still alive in 2011, living in Sweden and registered in LISA.

Gainfully employed individuals include those on temporary sickness benefits (sjukpenning) but not those on disability pensions (sjukersättning). Sickness benefits can be received for a longer period of time. After 3 months, and then again after 6 months, a person's health is examined against their work ability. Since in most cases it is economically better to receive sickness benefits than to take out pensions, we expect to be able to detect decreasing health that leads to retirement when looking at a distance to retirement in 3 months steps. Hence, we can draw a clear timeline whether health shocks appear before retirement or after. Individuals on disability pensions were excluded.

We define retirement as the transition from being gainfully employed without pension benefits to receiving only retirement benefits. Thereby, we exclude those who receive pensions but continue working. In 2009, 281,450 individuals had income fully based on paid work (excluding self-employed and individuals with very low income below the “price base amount” (in 2009: 42.800 SEK ~ 4.600 € p.a.); for a similar approach identifying different pathways into retirement (see König et al., 2021). While most of them (50.8%) were still fully working in 2011, 29,090 individuals (10.3%) reported that their entire income in 2011 was from pension payments. Precise information on the month of first pension receipt in 2010 was available for 25,133 individuals. Of these individuals, data on specialist visits was available for *N* = 25,122 and data on hospitalisation was available for all *N* = 25,133.

We clustered secondary healthcare use data into 3-month periods centred around the month of retirement [T0 = 0 (±1) month of retirement] to reduce empty cells without losing to much details in the temporal dynamics of the developments. In sum, we observed a period from 3 years and 1 month prior to 5 years and 1 month after retirement for a total of 33 observation points. As full-information was available, a balanced panel design with 829,026 observations on specialist visits and 829,389 observations for hospitalisation was built.

### Gender and Education

Gender was coded as either 0 = “male” or 1 = “female”. We defined three educational groups similar to a three-step ISCED: −1 = “low education” (elementary or less), 0 = “middle education” (middle vocational and vocational with A-level), and 1 = “high education” (higher vocational or tertiary education).

### Statistical Analyses

We conducted all analyses with Stata14. First, we compare mean levels using one-way analysis of variance to assess gender differences in education and secondary healthcare use at retirement (T0). We then apply Generalised Estimating Equations models (Liang and Zeger, 1986) with repeated measurements and autoregressive correlation structure. This type of multilevel model separates the variances within the individuals from the variance between individuals and therewith acknowledges that observations within an individual are more similar than those between individuals. As the outcome variables are binary, we apply a logistic distribution function to analyse secondary healthcare use during the retirement transition. The results are displayed as odds ratios (OR) with 95% confidence intervals (CI).

## Results

### Sample Characteristics and Gender Differences at Retirement

Just over half (54.3%) of the sample were women and 23.3% had a tertiary degree. The educational distribution differed significantly between men and women: 27.7% of the women vs. 18.1% of the men were highly educated. This is consistent with public statistics (OECD, 2019). Around the time of retirement, 16.4% of the sample visited a specialist and 0.9% spent at least 1 day in a hospital over a 3 months period. There were no gender differences in secondary healthcare use at retirement. Further sample characteristics and secondary healthcare use at retirement, overall and split by gender are provided in Table 1.

**Table 1:** Sample characteristics and secondary healthcare use at retirement in total numbers (and percent).

	**Total**	**Male**	**Female**	***p-value***
	25,133 (100.0)	11,488 (45.7)	13,645 (54.3)	
**Education**				
1. Low	7,272 (28.9)	3,890 (33.9)	3,382 (24.8)	
2. Middle	12,007 (47.8)	5,519 (48.0)	6,488 (47.5)	
3. High	5,854 (23.3)	2,079 (18.1)	3,775 (27.7)	0.00
**Specialist visits at T0 (N** **=** **25,122)**				
Specialist visit (yes)	4,113 (16.4)	1,879 (16.4)	2,234 (16.4)	0.98
**Hospitalisation at T0 (N** **=** **25,133)**				
Hospitalisation (yes)	215 (0.09)	104 (0.09)	101 (0.09)	0.43

*T0 represents the time of retirement transition. Gender differences were tested using one-way ANOVA*.

### Specialist Visits During the Retirement Transition

**Figure 1:**
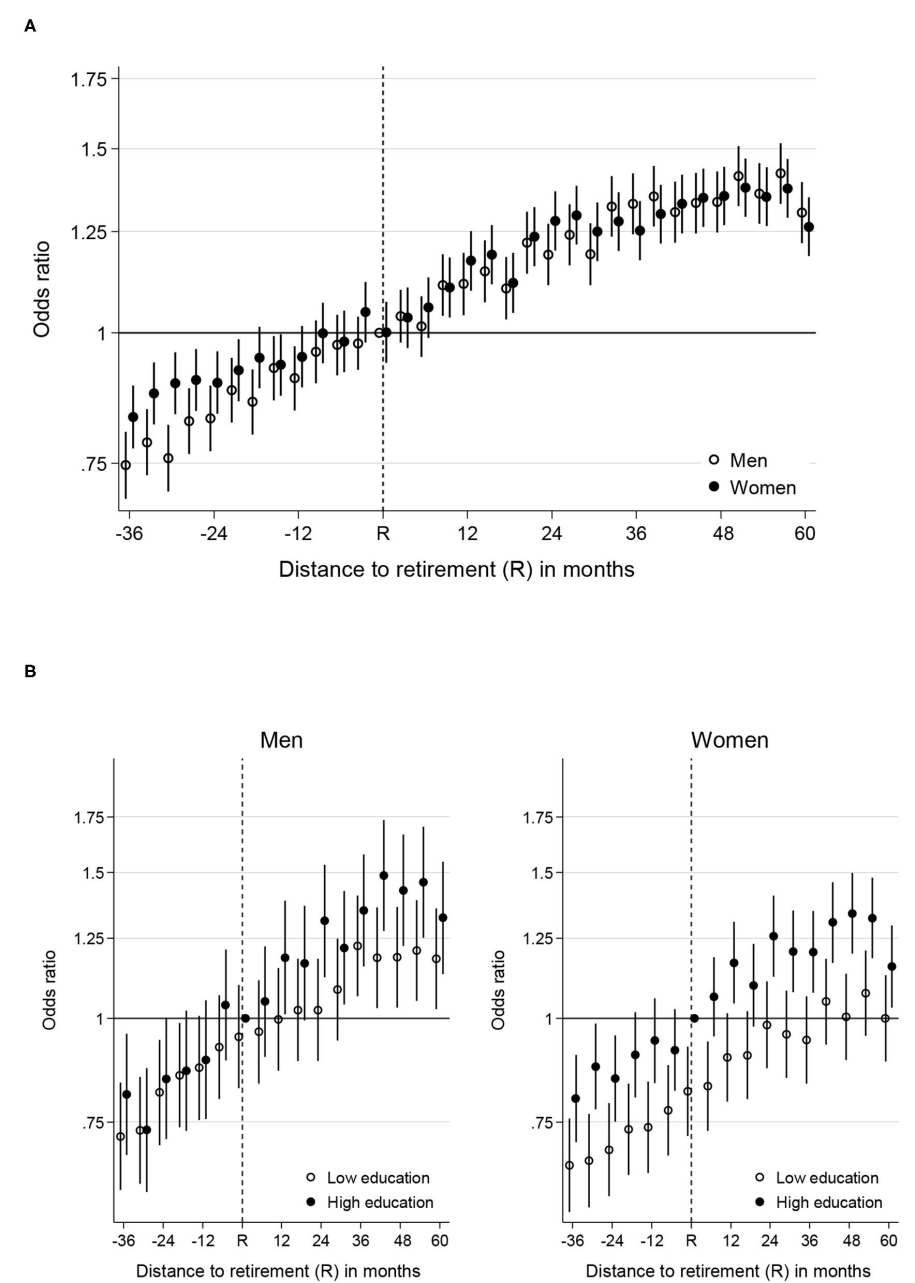
**(A)** Likelihood of specialist visit (expressed as odds ratio) during the retirement transition for men and women. Reference category is recently retired men. **(B)** Likelihood of specialist visit (expressed as odds ratio) during the retirement transition by gender and education. Reference category is recently retired high educated men or women. Only data from the low and high education groups and every second measurement occasion are displayed.

Figure 1A shows the OR for a specialist visit separately for men and women from 36 (±1) months before through 60 (±1) months after retirement. The odds ratio indicates the risk for a visit a specialist in a particular 3 months period compared to risk at the time of retirement for men. For instance, 36 months before retirement, the OR of 0.75 indicates that at that time men had a 25 percent lower risk of visiting a specialist than at the moment of retirement. For men, the OR for specialist visits increased more or less linearly from 0.75 (CI: 0.69–0.80) to 1.42 (at 57 months, CI: 1.33–1.51). For women, also a highly linear increase from 0.83 (CI: 0.78–0.89) to 1.37 (at 57 months, CI: 1.29–1.46) can be found. There was a small decline in the OR at the last observation [e.g., at 60 months men had an OR of 1.30 (CI: 1.22–1.39)]. There were no significant short-term changes in specialist visits either in the months before or after retirement.

In the years before retirement, women were more likely to visit a specialist than men [e.g., at −24 months, women had an OR of 0.90 (CI: 0.84–0.96) while men had an OR of 0.83 (CI: 0.77–0.89)]. The OR for specialist visits for women converged with the OR for men around 2 years before retirement, after which no significant gender differences were observed.

Figure 1B displays the change in OR for specialist visits for men and women split by educational level. The reference category is the higher educated again at time of retirement. To reduce complexity, we display only every second measurement occasion and only the OR for people with high and low education. The OR for people with middle education was always between the OR for people with high and low education. For men, there were no significant differences in the OR for specialist visits across educational groups until 12 months after retirement [low education: 1.00 (CI: 0.86–1.15); high education: 1.18 (CI: 1.01–1.39)]. In the 2 years following retirement, the differences between male educational groups increased 24 percentage points [low education: 0.95 (CI: 0.82–1.10) at T0 to 1.02 (CI: 0.89–1.33) at 24 months; high education: 1.00 (CI: not available because reference point) at T0 to 1.31 (CI: 1.12–1.53) at 24 months]. The increasing educational difference was primarily driven by a much larger increase in specialist visits among men with high education and a slower increase among men with low education.

In contrast to men, women with high education consistently had a higher OR for specialist visits than women with low education across the entire retirement transition. The difference between women with low and high education increased from 18 percentage points around the time of retirement to 28 percentage points at 24 months after retirement (low education: 0.82 (CI: 0.72–0.93) at T0 to 0.98 (CI: 0.87–1.11) at 24 months, high education: 1.00 (CI: not available because it is a reference point) at T0 to 1.26 (CI: 1.12–1.40) at 24 months). Hence, 2 years after retirement, the educational differences observed among men and women were of similar magnitude.

### Hospitalisation During the Retirement Transition

**Figure 2:**
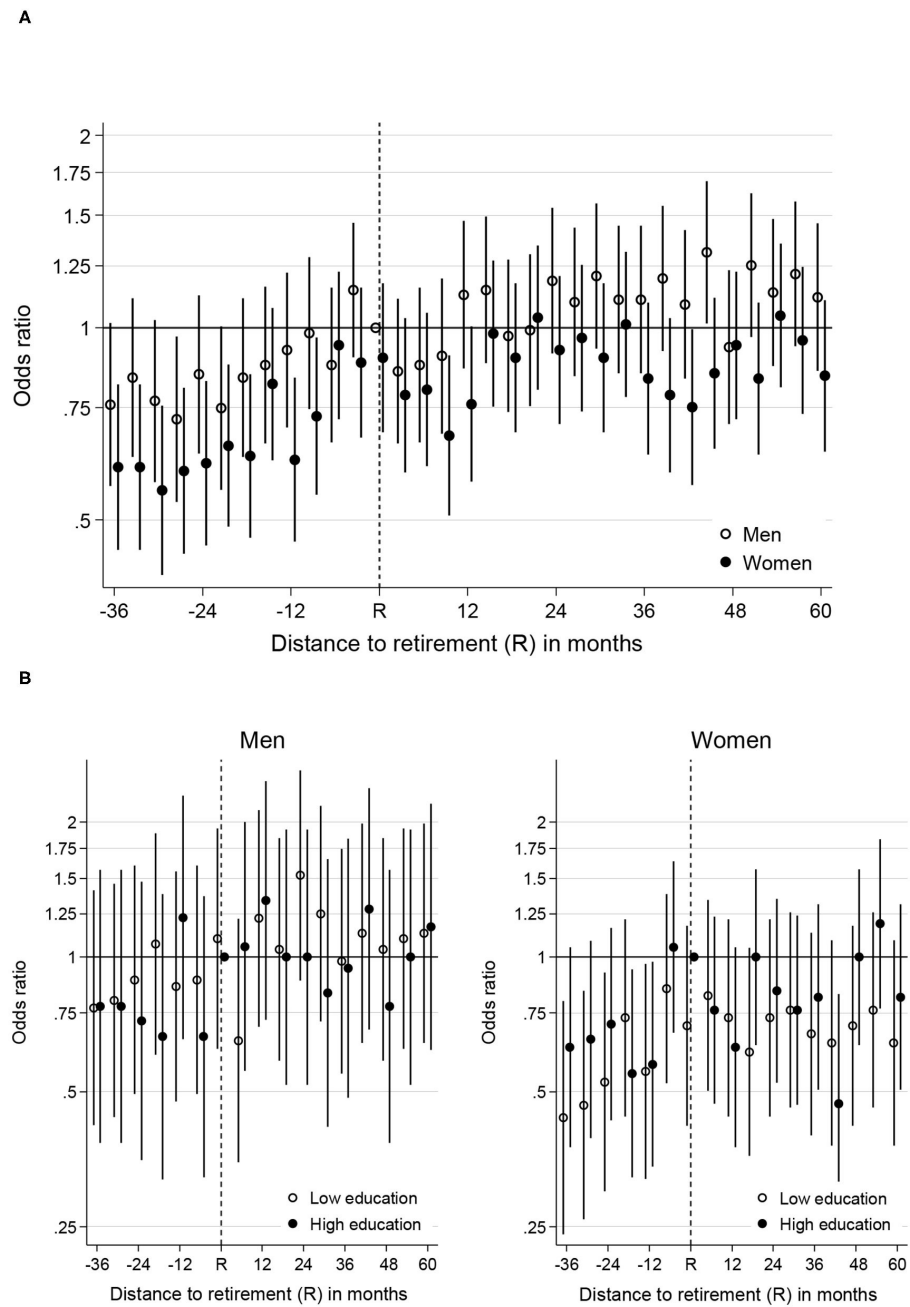
**(A)** Likelihood of hospitalisation (expressed as odds ratio) during the retirement transition for men and women. Reference category is recently retired men. **(B)** Likelihood of hospitalisation (expressed as odds ratio) over the retirement transition by gender and education. Reference category is recently retired high educated men or women. Only data from the low and high education groups and every second measurement occasion are displayed.

Figure 2A displays the OR for hospitalisation for men and women from 36 months before to 60 months after retirement. The OR for hospitalisation increased for both men [0.76 (CI: 0.57–1.02) to 1.21 at 57 months after retirement (CI: 0.94–1.58)] and women [0.60 (CI: 0.45–0.81) to 0.95 at 57 months (CI: 0.64–1.10)]. The OR at the last observation (60 months after retirement) was a bit lower than the previous observation but the difference was not statistically significant. For women, the OR for hospitalisation was lower 3 years to 1 year before retirement [−12 months: 0.62 (CI: 0.46–0.83)] than at retirement [0.90 (CI: 0.68–1.17)]. After retirement, the trend was less clear. For men, retirement did not seem to be associated with any changes in the OR for hospitalisation. Due to the increasing hospitalisation in the year before retirement for women, previously existing gender differences levelled out around retirement and there was no clear pattern after retirement. There were also no significant differences between educational groups among either men or women (see Figure 2B).

## Discussion

The current study uses Swedish register data and Generalised Estimating Equations models to estimate two types of secondary healthcare use during the retirement process for all individuals registered in Sweden born between 1943 and 1949 and retiring fully in 2010. Our main result is that we found no evidence that secondary healthcare use changed as in the short-term after retirement *per se* and is therewith in line with a list of previous studies on specialist visits (Bíró, 2016; Lucifora and Vigani, 2018; Nielsen, 2019) and hospitalisation (Coe and Zamarro, 2015; Eibich, 2015; Grøtting and Lillebø, 2020). We found that specialist visits increased gradually across the observation period, indicating an age as opposed to a retirement effect. We did not observe any overall changes in hospitalisation, nor did we find any gender and/or educational differences. Generally, the low prevalence of hospitalisation resulted in large CI and hence no statistically significant differences based on either gender or educational level.

Importantly, the current study found that gender differences in secondary healthcare use were most present in the years before retirement (i.e., while in paid work). Before retirement, women were more likely to visit a specialist and less likely to be hospitalised than men. This may be related to labour market stratified stressors and resources which are associated with jobs that typically men and women possess. The gender differences decreased (and disappeared) already in the last year. We therefore assume that labour market institutions lead to gendered health(care) risks and resources and that labour market institutions begin to lose their structuring power already in the year leading up to retirement, which might be due to anticipation. For instance, some individuals may delay non-critical specialists visits in anticipation of more flexibility in times of retirement. We observe this mainly among women. Three years prior to retirement, women might have better opportunities to visit specialists due to part-time work, compared to men. That might explain gender differences before retirement. One year prior to retirement, we observe this anticipation effect, where working hours may not be related to healthcare seeking behaviour. Since we do not observe this pattern for acute treatment in hospitals, we argue that the anticipation effect mainly concerns healthcare seeking behaviour, and not necessarily healthcare needs. This pattern might be specific to Sweden and countries with similar welfare regimes. In liberal countries, where healthcare provision is more closely linked to employment status, individuals might want to visit specialists before retirement.

Examining the intersection of gender and education revealed an interesting pattern with regard to specialist visits. Namely, women with high education were more likely to visit a specialist compared to women with low education over the entire period. The female educational difference increased after retirement. A difference in specialist visits between men with low and high education appeared only after retirement. Male and female educational differences were of similar magnitude after retirement. Taking these results together, education-based inequalities in secondary healthcare use seem to become more important after retirement. Education-based inequalities in a number of individual resources [e.g., income, leisure activities, social support (Wetzel et al., 2019), and subjective wellbeing (Wetzel et al., 2016)] also tend to increase after retirement.

The findings contribute to research regarding consequences of retirement and social policy research. They can be interpreted as indicator that retirement affects healthcare only little on average but that particular groups could profit from special attention. In the current study, lower SES was related with less advantageous changes with retirement. On the other side, gender differences declined with retirement. This might indicate that the labour market enforces gender differences while at the same time level-out SES differences (at least for those previously working). Moreover, this study contributes not only to the Swedish case but conclusion for other countries can be drawn. The Scandinavian Welfare state regime is known for their high level of redistribution aiming to reduce social inequalities—in particular between genders. Accordingly, in countries in which inequality receives less socio-political attention, both even larger educational and gender differences can be expected. With retirement transition, the current study would suggest that gender differences would decline also in mid- and larger inequality countries while educational differences might increase. While this generally points to an interesting research question, future research might want to address educational and gender-differences more explicitly. For example, Wetzel et al. (2019) found that inequality in number of chronic conditions increases by SES and gender without finding inequality in both dimensions before retirement—however only for those who have been previously unemployed. The current study would argue that declines in gender differences could be attributed to gender-segmentation of the labour market (e.g., positions of power, income, part-time) which become levelled out with retirement. For SES differences, retirement seems to be a life event leading to more (dis)advantageous developments, as previous research has found for other outcomes (e.g., Westerlund et al., 2009; Wetzel et al., 2016).

The current study has several strengths. We used detailed observational data from an entire birth cohort covering several years before and after retirement. We were therefore able to avoid problems associated with selectivity and observe anticipatory, short- and long-term effects of retirement. We used a precise definition of retirement as the end of gainful work and hence the time point at which, in particular, daily life routines and time structure drastically change. One reason why the literature on secondary healthcare use during retirement has been inconclusive may be because studies have considered many different retirement pathways [e.g., retirement from unemployment or disability pensions, bridge employment (Schmälzle et al., 2019; König et al., 2021)]. Finally, considering two indicators of secondary healthcare use, namely specialist visits and hospitalisation, allowed us to reveal how retirement affects different types of secondary healthcare.

Future research should address some of the limitations of the current study. We focused only on the secondary healthcare use of people retiring from paid work. Future research that considers primary and secondary healthcare as well as other pathways to retirement would contribute to a more comprehensive understanding. We did not differentiate the purpose of specialist visits (e.g., prevention vs. treatment), nor between different kinds of specialists, a limitation future research could address. Also, a comparison of several social inequality indicators might help to better the social stratification of (changes in) healthcare use. Finally, future research should examine the mechanisms by which the labour market affects social differences in secondary healthcare use.

## Conclusion

Our findings have important implications for individuals and societies. First, retirement *per se* does not appear to be a cause for concern with regard to secondary healthcare expenditures in Sweden. Second, how secondary healthcare use changes with retirement depends on gender and education: gender differences become smaller while educational differences become bigger post-retirement. These findings may indicate that the labour market institutions contribute to gender differences and partially suppress educational differences, effects which lose their power with retirement.

## Data Availability Statement

The used datasets are available for research purposes after ethical approval. Both applied registries are administered by Statistics Sweden. The database was conceived, compiled and made available by the Division Ageing and Social Change (ASC) at Linköping University, Sweden, within the project Ageing in a Changing Society (PI: Prof. Andreas Motel-Klingebiel). Code for data handling and statistical analysis is available on OSF [https://osf.io/ax3ec/?view_only=f549aa85424244529e2bbfea4e577212 (This is link is anonymized for review process)].

## Ethics Statement

Ethical approval for record-linkage of the register data was obtained from Linköping Regional Ethical Review Board (Dnr 2016/293-31).

## Author Contributions

MW had the initial idea, did the data management with support by SKe, performed the statistical analysis, and wrote the first draft of the manuscript. SKö and SKe revised the first draft. All authors contributed to conception and design of the study. All authors contributed to manuscript revision, read, and approved the submitted version.

## Funding

Major parts of this work originated during a research stay of MW at the Linköping University (Sweden) which was supported by a mobility grant of the Excellence Initiative of University of Cologne.

## Conflict of Interest

The authors declare that the research was conducted in the absence of any commercial or financial relationships that could be construed as a potential conflict of interest.

## Publisher's Note

All claims expressed in this article are solely those of the authors and do not necessarily represent those of their affiliated organizations, or those of the publisher, the editors and the reviewers. Any product that may be evaluated in this article, or claim that may be made by its manufacturer, is not guaranteed or endorsed by the publisher.

## Footnotes

^1^We decided to keep the retirement period (P) fixed to focus on similar living conditions and retirement regulations for the observed individuals. Although the retirement age (A) range seems to vary significantly, statistics Sweden showed that retirement age does differ only marginally between gender and educational groups (Statistics Sweden, 2013).
